# Pseudohypoparathyroidism mimicking cervical diffuse idiopathic skeletal hyperostosis with dysphagia: A case report and literature review

**DOI:** 10.1016/j.bonr.2021.101111

**Published:** 2021-07-27

**Authors:** Netanja I. Harlianto, Firdaus A.A. Mohamed Hoesein, Pim A. de Jong, Jorrit-Jan Verlaan, Jan Westerink

**Affiliations:** a Department of Orthopedic Surgery, University Medical Centre Utrecht, Utrecht, the Netherlands; b Department of Radiology, University Medical Centre Utrecht, Utrecht, the Netherlands; c Department of Vascular Medicine, University Medical Centre Utrecht, Utrecht, the Netherlands

**Keywords:** Case report, Dysphagia, Osteophytes, Diffuse idiopathic skeletal hyperostosis, Pseudohypoparathyroidism

## Abstract

Dysphagia due to extensive ossification at anterior segments of the cervical spine is a rare occurrence and is usually attributable to diffuse idiopathic skeletal hyperostosis (DISH).

We present the case of a 74-year-old female with dysphagia most likely due to ossification in pseudohypoparathyroidism type 1a (PHP1a). PHP1a is a rare, autosomal dominant disorder caused by mutations in the GNAS1 gene. Our patient had characteristic phenotype features of PHP1a, also known as Albright's hereditary osteodystrophy (AHO), which was diagnosed without genetic confirmation.

She was conservatively treated with dietary measures and observation, and reported persisting symptoms of dysphagia at six-month follow-up. This is the first case to describe dysphagia in PHP1a with a similar presentation to DISH.

## 1 Background

Osteophytes at the anterior cervical spine are often observed but rarely known to cause symptomatic compression of esophagus and/or trachea, with dysphagia and/or airway obstruction as result (Verlaan et al., 2011). Diffuse idiopathic skeletal hyperostosis (DISH) is the most common cause of osteophytes at the cervical level with 204 cases of dysphagia and airway obstruction reported between 1980 and 2009. We present a unique case of a patient with dysphagia most likely due to ossification in pseudohypoparathyroidism type 1a (PHP1a), which, to our best knowledge, has not been previously described in the literature. The patient provided informed consent for the publication of her data.

## 2 Case report

A 74-year-old female patient was referred to our institution by her general practitioner due to complaints of chronic neck pain and a limited range of motion for several years. Moreover, she reported difficulty in swallowing solid foods which started 9 months earlier, and a weight loss of 5 kg. She reported no symptoms of snoring or sleep apnea. Her medical history included obesity, hypertension, dyslipidemia, chronic renal insufficiency, coronary artery disease, hypothyroidism, and osteoporosis. She had no history of cervical trauma or surgery. She was previously seen in the internal medicine outpatient clinic because of PHP1a with Albright's hereditary osteodystrophy (AHO) (diagnosed in 1988) and type 2 diabetes mellitus (diagnosed in 1997). During physical examination in 1988, a height of 1.52 m, a weight of 105 kg, and a body mass index of 45.5 kg/m^2^ were observed. Relevant lab results performed in 1988 are shown in Table 1, and included elevated levels of parathyroid hormone (PTH) and a serum calcium on the lower end of normal. Although it is unknown when her first symptoms of hypocalcemia started, in the years prior to the established diagnosis of PHP, she frequently had complaints of paresthesia and muscle spasms in her right hand with intermitting neck pain.

**Table 1:** Relevant laboratory measurements at diagnosis and current presentation.

	Diagnosis in 1988	Current presentation	Normal range
25OHD (nmol/L)	19.4	89	50–100 nmol/L
1,25(OH)2D (pmol/L)	30.6	NA	50–170 pmol/L
Calcium (mmol/L)	2.20	2.34	2.2–2.6 mmol/L
Phosphate (mmol/L)	1.26	1.28	0.8–1.5 mmol/L
PTH (pmoll)	10.5	12	1.0–7.0 pmol/L
Creatinine (μmol/L)	70	144	49–90 μmol/L

25OHD: 25-hydroxyvitamin D; 1,25(OH)2D: 1,25-dihydroxyvitamin D; PTH: parathyroid hormone; NA: not available.

Clinical features that supported the diagnosis of PHP1a in 1988 with AHO included the patients' hereditary obesity and physical features such as a round face, low nasal bridge, and short nose and neck. Because of the clear clinical depiction of her symptoms related to AHO, our patient did not undergo genetic testing. The diagnosis PHP was established after the administration of intravenous PTH (200 IU), in which no increase of 3′,5′-cyclic adenosine monophosphate (cAMP) levels in serum and urine, or urine phosphate levels were observed. The patient had a daughter who also received injections of PTH, where in contrast, levels of cAMP increased in both serum and urine after PTH administration. Thus, no PHP was diagnosed in her daughter. Furthermore, she had two other sons who were also not diagnosed with PHP.

**Fig. 1:**
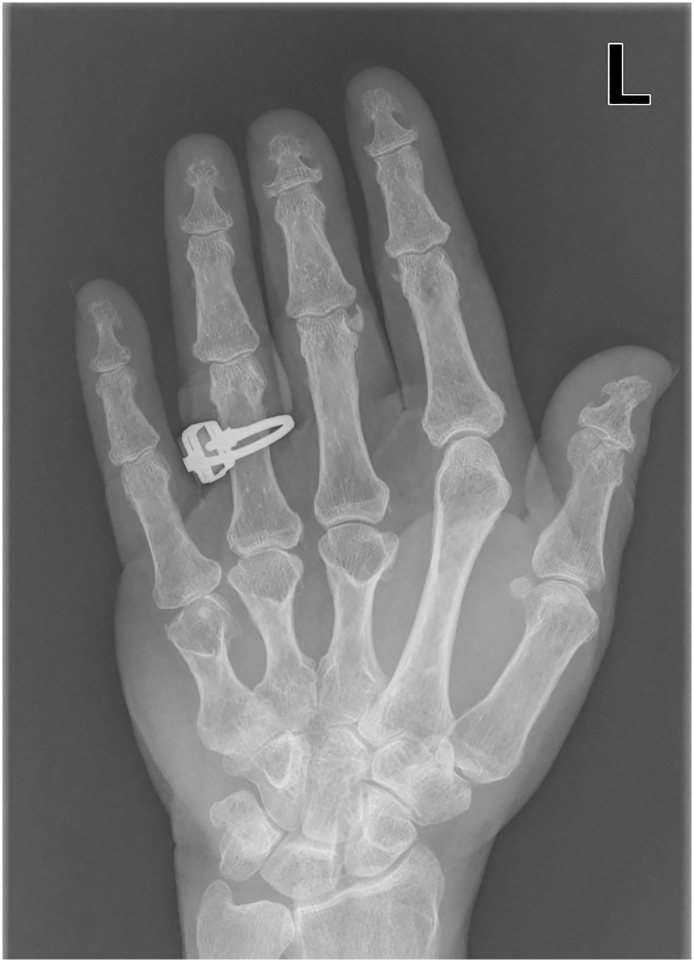
Radiography of the left hand with evident brachydactyly of metacarpals III-V.

Since diagnosis our patient had been taking calcitriol (0.25 micrograms each day) for her PHP1a, which was later replaced by combined treatment of calcium carbonate and cholecalciferol in 2012 (current dosage: 1000 mg/880 I.E.). Hand radiography was performed in 2004, which displayed shortened phalanges and evident shortening of the metacarpals III-V in both hands (Fig. 1).

**Fig. 2:**
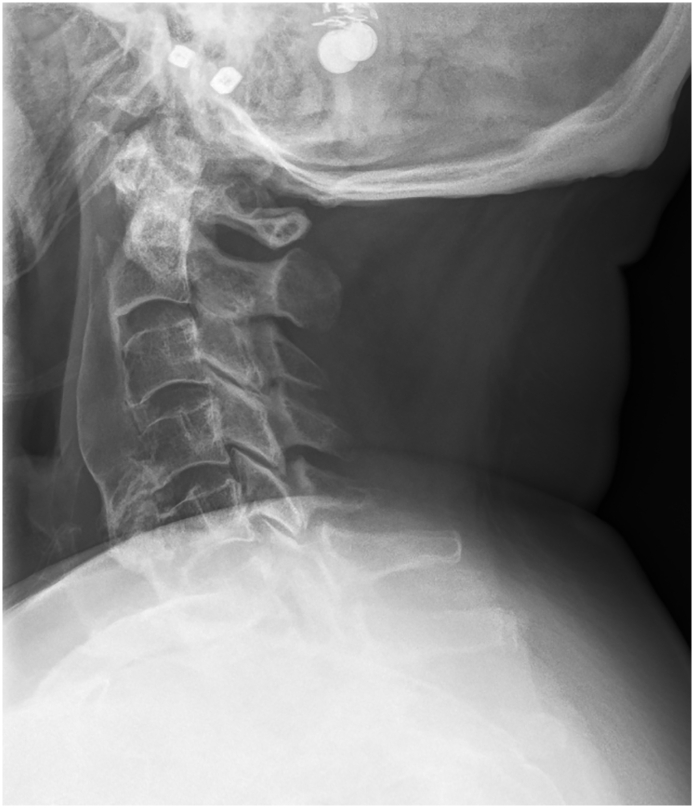
Anterolateral ossification with bone growth from cervical levels C2-C5.

On current evaluation, lab results confirmed the presence of metabolic hyperkalemia, with elevated levels of PTH, and normal 25-OH-vitamin D levels (Table 1). She was classified with Type G3b renal insufficiency in 2009, with the most recent estimated glomerular filtration rate value of 39 mL/min/1.73m^2^. Electrocardiogram revealed a sinus rhythm of 63/min and narrow QRS morphology with prolonged QTc time (482 ms). Lateral radiographs of the cervical spine (Fig. 2) displayed flowing ossifications near the anterior longitudinal ligament from C2-C5, with preserved intervertebral disc space. The largest measured osteophyte thickness was 15 mm at C4-C5, which most likely caused impingement of the esophagus. Radiographs of the thoracic spine did not show any ectopic ossifications.

Both the presence of risk factors for DISH, such as the patients' age, the presence and duration of type 2 diabetes, obesity, and hypertension, as well as her PHP1a were considered as causal factors in the development of the cervical osteophytes leading to symptoms of dysphagia. Our patient stated that the symptoms of dysphagia were tolerable and that it could be partly alleviated with appropriate dietary measures, thus surgical treatment was not initiated. She reported persisting symptoms of neck pain and dysphagia at six-month follow-up.

## 3 Discussion

PHP1a is an autosomal dominant disorder caused by mutations in the GNAS1 gene, which is primarily characterized by end organ dysfunction to PTH, leading to reduced levels of calcium and activated vitamin D, and elevated levels of both PTH and phosphate. AHO is the phenotypic presentation of PHP1a. Patients may suffer from learning impairments and distinct musculoskeletal features related to PHP such as obesity, short stature, brachydactyly, and facial features like a small nose, small nasal bridge, and round face. Moreover, resistance to other hormones is sometimes observed in PHP due to loss of function of GNAS. (Wilson and Hall, 2002; Mantovani et al., 2018). For instance, our patient also suffered from resistance to thyroid stimulating hormone alongside her resistance to PTH, which is frequently seen in PHP1a (Wilson and Hall, 2002; Mantovani et al., 2018).

Ossification in AHO is usually limited to incidental findings most often found in the soft tissues. Patients with PHP1a usually have disharmonic or advanced bone age, and cases of ankylosis and joint deformation have also been reported in young PHP1a patients. Ectopic ossifications are a manifestation of Gsα deficiency in mesenchymal stem cells, with de novo formation of extraskeletal osteoblasts that form islands of ectopic bone in the dermis and the subcutaneous fat (Mantovani et al., 2018). The long term dysfunction of the PTH-pathway results in hyperphosphatemia and hypocalcemia, which may instead cause ectopic calcifications in PHP1a (Wilson and Hall, 2002; Mantovani et al., 2018).

The treatment of PHP1a is aimed at maintaining normal serum levels of calcium and phosphate, thus preventing secondary complications such as intracranial depositions of calcium (which would lead to Fahr syndrome), or calcium accumulation in the eyes or kidneys (Mantovani et al., 2018). Though diagnosis in our patient was established with intravenous injections of PTH, the most recent consensus statement recommends that molecular genetic analysis should be performed (also necessary for genetic counselling), complementary to the clinical and laboratory features in PHP (Mantovani et al., 2018). Our patient's offspring had no symptoms of PHP, most likely due to the imprinting nature of the GNAS gene, which has an impact on the clinical outcome (Mantovani et al., 2018).

DISH, in contrast, is a common systemic ankylosing condition characterized by ossifying enthesopathies and hyperostosis at the anterolateral aspect of the spine. DISH mostly affects older male individuals, increases in prevalence with age as a prevalence of up to 42% has been reported in patients over the age of 65 (Holton et al., 2011; Mader et al., 2013). DISH is classified using the Resnick criteria (Resnick and Niwayama, 1976) which includes: anterolateral ossification of at least four vertebral bodies; with preserved intervertebral disc height; and absence of apophyseal joint erosion or sacroiliac inflammatory changes. Although the exact pathophysiology of DISH remains undetermined to date, metabolic factors with low-grade inflammation are thought to contribute to ossification in the disease as DISH has been frequently associated with hyperinsulinemia, type 2 diabetes, obesity, and hypertension (Mader et al., 2013; Mader et al., 2021; Harlianto et al., 2021). Furthermore, intracerebral and coronary calcifications have also been reported to be increased in patients with DISH (Mader et al., 2013; Oudkerk et al., 2019).

When reviewing the literature for comparable cases of symptomatic PHP with a radiological presentation similar to DISH, we identified two cases. Both cases were at the level of lumbar spine with symptoms of back pain, whereas one case resulted in myelopathy and spinal cord compression (Jiang et al., 2010; Mak and Mok, 2005). Likewise, in two other patients with idiopathic hypoparathyroidism, a clinical presentation comparable to DISH was reported (Unverdi et al., 2009; Lambert and Becker, 1989).

Other paravertebral ossifications, not limited to the anterior longitudinal ligament of the spine, have been reported for both PHP and hypoparathyroidism, including ossification of the posterior longitudinal ligament and the ligamentum flavum (Firooznia et al., 1985; Sohail et al., 2018; Jeon et al., 2019). In some cases these ossifications have even resulted in spinal cord compression with progressive paraparesis or spastic tetraparesis (Firooznia et al., 1985; Iwase et al., 2002).

As both idiopathic hypoparathyroidism and PHP with AHO are characterized by hypocalcemia and hyperphosphatemia, an overlap in symptoms can be observed in both disorders. For the described patients, the age of onset of symptoms was usually under 50 years. The development of ossification of the paravertebral ligaments in patients with PHP or idiopathic hypoparathyroidism can be a result of both suppressed PTHR1 signaling (or GNAS mediated signaling) and chronic hypocalcemia with concomitant hyperphosphatemia [21]. Our patient started pharmacological treatment for PHP at the age of 42, and was thus untreated for a large portion of her life.

We acknowledge that the concurrent presence of DISH alongside ectopic ossification of PHP1a cannot be established with certainty. Another limitation of our report is the lack of genetic data for our described patient. Nevertheless, herein we report the first case of dysphagia in a PHP1a patient with distinctive AHO characteristics, similar in presentation to DISH. PHP/hypoparathyroidism can result in spinal ossification, which may cause neurological symptoms or dysphagia. In rare instances, PTH-pathway abnormalities should be considered in the differential workup of DISH.

## Funding

None.

## Author contributions

**Netanja I. Harlianto:** Conceptualization, Data curation, Writing, review & editing. **Firdaus A.A. Mohamed Hoesein:** Writing, review & editing. **Pim A. de Jong:** Writing, review & editing. **Jorrit-Jan Verlaan:** Writing, review & editing. **Jan Westerink:** Conceptualization, Data curation, Supervision, Writing, review & editing.

## Declaration of competing interest

All authors have completed the ICMJE uniform disclosure form at www.icmje.org/coi_disclosure.pdf and declare: no support from any organization for the submitted work; no financial relationships with any organizations that might have an interest in the submitted work; no other relationships or activities that could appear to have influenced the submitted work; PJ received consulting fees from Sanfit and Inozyme.
